# Enrichment Free qPCR for Rapid Identification and Quantification of *Campylobacter jejuni*, *C. coli*, *C. lari*, and *C. upsaliensis* in Chicken Meat Samples by a New Couple of Primers

**DOI:** 10.3390/foods10102341

**Published:** 2021-09-30

**Authors:** Priya Vizzini, Jasmina Vidic, Marisa Manzano

**Affiliations:** 1 Dipartimento di Scienze AgroAlimentari, Ambientali e Animali, Università di Udine, 33100 Udine, Italy; vizzini.priya@spes.uniud.it; 2 AgroParisTech, INRAE, Micalis Institute, Université Paris-Saclay, 78350 Jouy en Josas, France; jasmina.vidic@inrae.fr

**Keywords:** *Campylobacter* spp., qPCR, spiked chicken meat, foodborne pathogens

## Abstract

*Campylobacter* is the main cause of bacterial foodborne disease and poultry meat is the principal source of human infections. Rapid methods for *Campylobacter* detection are urgently needed to decrease high bacterial prevalence in poultry products. In this study, we developed new primers, CampyPFw and CampyPRv, that target the 16S-23S rRNA genes of *Campylobacter jejuni*, *C. coli*, *C. lari* and *C. upsaliensis.* The primers were tested on positive and negative reference strains in pure cultures and in inoculated poultry meat samples before their application in real-time PCR (qPCR) protocol for analyzing chicken meat samples. In parallel, the samples were tested by using the ISO 10272-1:2006 method. The qPCR protocol based on CampyPFw and CampyPRv showed good sensitivity, with the limit of detection of 4.6 × 10^2^ cells/mL in chicken samples without enrichment steps.

## 1. Introduction

The thermotolerant *Campylobacter* species, especially *C. jejuni* and *C. coli*, are the leading cause of campylobacteriosis, the zoonotic enteric infection for which its incidence has increased in both developed and developing countries in the last 10 years [1]. *Campylobacter* spp., the major zoonotic disease agent since 2005 [2], may cause gastroenteritis, severe septicemia bloodstream infection, inflammatory bowel disease, reactive arthritis, and Guillain-Barreé syndrome [2]. The most important source of campylobacteriosis in humans is raw or insufficiently cooked chicken meat as well as cross-contamination while handling meat contaminated with *Campylobacter* spp.

*Campylobacter* shows more than 75% prevalence in the EU member states in broiler meat [3,4]. Currently, samples from broiler meat and skin are analyzed by using labor- consuming and time-consuming enumeration processes that are mostly based on viable plate count methods [1]. The official culture-based methods are not suitable for routine analysis, because they provide results in 5–7 days while most of the poultry-based products are consumed within a few days. In addition, *Campylobacter* is a fastidious organism that loses its cultivability when poultry meat is stored at 4 °C or under oxygen, and, thus, cannot be detected by a plate count method [5,6,7].

Methods including microscopy and assays for the detection of metabolic activities, such as membrane potential, are sensitive and rapid but expensive and time-consuming [1,8,9]. Molecular methods, such as real-time PCR (qPCR), provide advantages in *Campylobacter* quantification, especially in terms of the turnaround time, specificity, and sensitivity [10,11]. The implementation of molecular techniques will enable rapid and accurate routine analysis, thereby preventing and/or reducing outbreaks in humans and improving our knowledge on *Campylobacter* contamination. qPCR has already been used for different purposes related to the poultry industry, including quantification in poultry carcass rinses [12,13,14,15], chilled or frozen carcass [16], fecal and cecal samples [17,18], carcasses [19], neck-skin [20,21], and samples from slaughterhouses [19,22]. Nevertheless, different steps in qPCR still have to be optimized before it can be widely accepted for identification and quantification of *Campylobacter* in poultry samples with low contamination levels. These limitations are mainly related to the method used for the extraction of DNA in various food matrices, the elimination of matrix inhibitors, and the detection and quantification of low number of cells per gram of foods.

In this study, a new couple of primers targeting the 16S-23S rRNA gene of the most prevalent *Campylobacter* spp., i.e., *C. jejuni*, *C. coli*, *C. lari*, and *C. upsaliensis*, was designed, tested, and used in qPCR for *Campylobacter* quantification. The qPCR assay was applied on both artificially and naturally contaminated chicken meat samples before the enrichment step (DNA extracted from the homogenization bag) and after the 48 h enrichment in Bolton broth (selective medium for the *Campylobacter* species). The results were compared with those obtained by using the plate count method and the standard method.

## 2. Materials and Methods

### 2.1. Microorganisms

The bacterial strains used in this study are listed in Table S1. All *Campylobacter* spp., *Helicobacter* spp., and *Arcobacter butzleri* strains were cultivated under specific microaerophilic conditions (6% O_2_, 7% CO_2_, 7% H_2,_ and 80% N_2_) generated by using an Oxoid™ CampyGen™ 2.5 L sachet (Thermo Fisher Scientific Inc., Milan, Italy). The revitalization procedure of cultures stored at −80 °C was conducted at 37 °C for 48 h in brain-heart infusion (BHI) broth (Thermo Fisher Scientific Inc., Milan, Italy). Furthermore, the *Campylobacter* isolates were incubated in Columbia blood agar base (Thermo Fisher Scientific Inc., Milan, Italy) supplemented with 5% *v*/*v* of sheep defibrinated blood. Pure colonies were isolated on BHI agar medium and subjected to Gram staining, oxidase and catalase tests, and cell-morphology analyses. Strains used as negative controls were cultivated on BHI agar medium at 30 or 37 °C for 24 or 48 h based on the optimum growth conditions of the microorganism, with the exception of *Lactobacillus plantarum* which required microaerophilic conditions. All bacteria were examined using Gram staining and cell-morphology analysis. Selective media PALCAM Agar Base, X.L.D. Agar, and Brilliance™ Bacillus Cereus Agar Base for *Listeria*, *Salmonella*, and *Bacillus cereus*, respectively, were purchased from Oxoid (Thermo Fisher Scientific Inc., Milan, Italy).

### 2.2. Chicken Samples and Plate Count Enumeration

Twenty chicken meat samples (named CC) purchased from local butcher shops in Italy in 2016 where they were conserved at 4 °C until purchase, were analyzed for bacterial enumeration by using the plate-count method and tryptone soya agar (TSA) at 30 °C for 48 h. Yeasts and molds were enumerated on malt extract agar supplemented with 10 μg/mL tetracycline (AMT) at 30 °C for 48 h; Enterobacteriaceae, were enumerated on violet red bile glucose agar (VRBGA) at 37 °C for 24 h; and *E. coli* and coliforms were enumerated on Coli-ID agar (Biomeriaux, Firenze, Italy) at 37 °C for 24 h.

Additionally, 10 g of each chicken sample (including skin and meat) was transferred to a Stomacher filter bag containing 90 mL of Bolton broth (Thermo Fisher Scientific Inc., Milan, Italy) and subjected to the ISO 10272-1:2006 method for *Campylobacter* spp. detection.

For the sake of confirmation, suspected colonies from each plate were streaked on blood agar base plates, half of each colony was incubated at 41.5 °C, and the other half was incubated at 25 °C for 48 h. Bacteria were subjected to oxidase tests and motility tests, that were carried out in Brucella broth (Thermo Fisher Scientific Inc., Milan, Italy) in order to verify the presence of the typical corkscrew-like movement used for *Campylobacter* spp. identification.

Serial decimal dilutions of *C. jejuni* overnight culture (BHI, microaerophilic conditions, 20 h) containing approximately 10^8^ cells/mL were inoculated into meat samples (named SC) to reach final concentrations of 10^7^, 10^5^, 10^3^, and 0 cell of *C. jejuni* per g of meat.

The spiked chicken samples were analyzed by colony count on mCCDA to confirm the inoculum, and DNA was extracted and used in a qPCR assay with the new primers.

**Figure 1:**
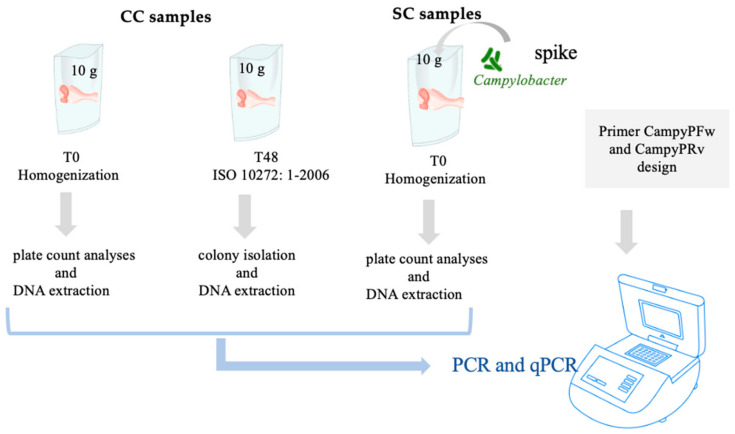
Workflow of the work performed for the detection of *Campylobacter* spp. in chicken samples.

The workflow of the procedure used in this work is reported in Figure 1.

### 2.3. DNA Extraction

DNA extractions from CC samples were carried at t_0_ (CCt_0_) and after enrichment at 48 h (CCt_48_), while DNA was extracted from SC samples (spiked samples) at t_0_ (SCt_0_). Both DNA extractions were performed following a previously published protocol [23]: Two mL was collected from the Stomacher bags containing SPW at t_0_ (SC and CC samples), and 2 mL was collected from the Bolton broth after 48 h (CC_t48_ samples). After centrifugation at 14,000× *g* for 10 min, the pellet was resuspended in 300 µL of breaking buffer (2% Triton X-100, 1% SDS, 100 mm NaCl, 10 mm Tris pH 8, and 1 mm EDTA pH 8) and 300 mL of phenol–chloroform–isoamyl alcohol 25:24:1 (Sigma, Milan, Italy) was added [24] with glass beads.

The cells were then homogenized in a bead beater (Mini-Bead Beater 8t, Biospec Products Inc., Bartlesville, OK, USA) three times, each for 30 s at maximum speed at room temperature. The amount of 300 mL of TE (10 mm Tris, 1 mm EDTA pH 7.6) was added, and the tubes were centrifuged at 12,000× *g* for 10 min at 4 °C. The aqueous phase was collected, and DNA was precipitated with 1 mL ice-cold absolute ethanol. After centrifugation at 14,000× *g* for 10 min at 4 °C, the pellet was dried under vacuum at room temperature and resuspended in 50 mL of sterile distilled water containing 2 IU DNase-free RNase (Roche Diagnostics, Milan, Italy). The samples were then incubated at 37 °C for 30 min before storage at −20 °C.

DNA concentration and purity were measured using a spectrophotometer (NanoDrop, ThermoFisher Scientific Inc, Milano, Italy). The extracted DNAs were used for qPCR.

### 2.4. Primer Design

New primers CampyPFw (5′-CTTTGCACGCAGGAGGTCA-3′) and CampyPRv (5′-ATGGTGGGCCTAACAAGACT-3′) were designed in the 16S-23S gene sequences GQ167702.1 of *C. jejuni*; GQ167720.1 of *C. coli*; AB644222.1 of *C. lari*; and DQ871249.1 of *C. upsaliensis* downloaded from GenBank (http://www.ncbi.nlm.nih.gov/genbank/). The software (http://multalin.toulouse.inra.fr/multalin/) for multiple sequence alignment with hierarchical clustering [25] AmplifX 1.7.0, OligoAnalyzer 3.1 (https://eu.idtdna.com/calc/analyzer) and FastPCR6.1 were used to verify the specificity of various bacteria belonging to both the same and different genera and animal gene sequences (Table S2), as previously described [26].

### 2.5. PCR and qPCR Protocols

CampyPFw and CampyPRv primers were tested for specificity by using end-point PCR. The reaction mixture contained the following reagents: 5 µL AmpliTaq buffer, 1.5 mM MgCl_2_, 1 µL dNTPs (10 mM of each dNTP), 1 µL of each primer (10 μM), 0.25 μL AmpliTaq DNA polymerase (5 units/µL), and 1 µL of DNA at 100 ng/µL. All reagents were purchased from Applied Biosystems (ThermoFisher Scientific Inc., Milan, Italy). In each assay, a negative control where the template DNA was replaced with an equal volume of nuclease-free water (NCT) was included. A thermal cycler C1000 Touch^TM^ (Bio-Rad Laboratories Inc., Hercules, CA, USA) was used.

Amplification conditions were as follows: denaturation at 95 °C for 5 min; 30 cycles of denaturation at 95 °C for 1 min; annealing at 58 °C for 30 s; extension at 72 °C for 30 s; and a final extension at 72 °C for 7 min. The PCR products were electrophoresed on a 1.5% agarose gel and visualized using ethidium bromide (Sigma-Aldrich Inc., Milan, Italy) at a final concentration of 0.5 μg/mL in a GeneGenius BioImaging System (Syngene Ltd., Cambridge, UK). The electrophoretic run was carried out at 120 V for 40 min.

A qPCR protocol with CampyPFw and CampyPRv was optimized by using a Rotor-gene Q thermocycler (Qiagen Inc., Milan, Italy). Calibration curves were performed using both serial dilutions of DNA in the range 10 ng/μL–100 fg/μL and concentrations of *C. jejuni* DSM 4688 cells from 10^8^ to 10 cell/mL. The PCR mixture contained the following reagents: 10 µL of SsoFast™ EvaGreen Supermix (2×) (Bio-Rad Laboratories Inc., Hercules, CA, USA), 1 µL of each primer (CampyPFw and CampyPRv) at 10 μM, and 1 µL of DNA template in a final volume of 20 µL. DNAs extracted from chicken samples SCt_0_, CCt_0_, and CCt_48_ were used; in each assay, a negative control was included.

The program consisted of hot-start activation at 98 °C for 2 min, 35 cycles of denaturation at 98 °C for 5 min, and annealing/extension at 60 °C for 20 s. Following a melting temperature analysis, a gradual increase in temperature from 60 to 95 °C (0.5 °C/5 s) was performed.

An end-point PCR was performed for samples 2CC and 3CC and *C. jejuni* DSM4688 (as reference) using primers P1V1 and P4V3 [27], purified using the QIAquick PCR Purification Kit (Qiagen Inc., Milan, Italy), and sent to Eurofins Genomics Co. (Ebersberg, Germany) for sequencing. The obtained sequences were processed in BLAST [28] to confirm *Campylobacter* identification.

## 3. Results and Discussion

### 3.1. Microbiological Analysis of Samples

The enumerations of the total viable counts of *Enterobacteriaceae*, coliforms, *E. coli*, yeasts, and molds obtained for 20 chicken samples, using the plate count method are reported in Table 1.

The total bacterial count in CC samples ranged from 1.3 × 10^4^ to 5.1 × 10^9^ CFU/g, with the exception of the 17 CC sample which showed values below 50 CFU/g (the limit of detection of the method used). Similarly, *Enterobacteriaceae* count ranged from 2.1 × 10^2^ to 2.4 × 10^4^ CFU/g; coliform values ranged from 1.3 × 10^1^ to 3.2 × 10^4^ CFU/g, except for samples 12 CC and 17 CC, which showed values below the limit of detection of the method used. *E. coli* values ranged from 2 × 10^1^ to 5.3 × 10^3^ CFU/g, except for the 19 CC sample, which showed a value below the limit of detection. Yeasts ranged from 1.7 × 10^2^ to 6.3 × 10^5^, except for the 17 CC sample, while showed that molds were below the limit of detection in all samples.

The total viable count for mesophilic microorganisms was acceptable for all chicken samples analyzed, except for 18 CC, 19 CC, and 20 CC, which were 2–3 log higher. Data obtained were in accordance with values reported in the guidelines of Piemonte Region, which are based on risk analysis in approved food microbiology. For fresh and refrigerated meat values of total viable count, mesophilic microorganisms from 10^6^ to 10^7^ CFU/g, Enterobacteriaceae from 10^4^ to 10^6^ CFU/g and *E. coli* from 10^3^ to 10^4^ CFU/g are considered acceptable.

Samples 2 CC, 3 CC, 8 CC, 10 CC, 16 CC, and 17 CC were positive for the presence of *Campylobacter* spp. based on the results obtained by the ISO 10272-1:2006 method after 4–6 h at 37 °C and 40–48 h incubation in Bolton broth (Table 2). No correlation was observed between total bacterial count and the presence of *Campylobacter* spp., which is in agreement with previously published results [29].

### 3.2. PCR and qPCR Analysis

**Figure 2:**
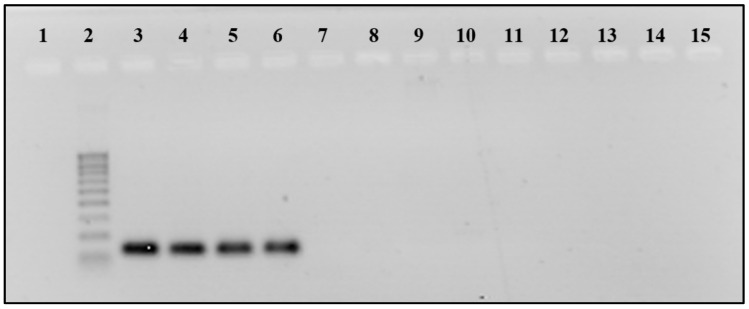
Specificity test with CampyPFW-CampyPRW at 58 °C annealing temperature. Line 1:-; Line 2: 100 bp DNA Ladder (Sigma. Inc., Milan, Italy); line 3: *Campylobacter jejuni* DSM 4688; line 4: *C. coli* DSM 24155; line 5: *C. lari* DSM 11375; line 6: *C. upsaliensis* DSM 5365; line 7: *C. fetus* DSM 5361; line 8: *Helicobacter suis* DSM 19735; line 9: *H. pylori* DSM 7492; line 10: *H. pylori* ICSS; line 11: *Arcobacter butzleri* DSM 8739; line 12: *Bacillus cereus* DI4A RC3; line 13: *Escherichia coli* DISTAM; line 14: *Lactobacillus plantarum* ATCC RAA 793; line 15: *Saccharomyces cerevisiae* ATCC 36024.

New CampyPFw and CampyPRv primers were tested using the end point PCR on DNAs extracted from the bacteria listed in Table S1 before their utilization in qPCR. Only *Campylobacter* strains produced the expected amplicons of 132 bp, confirming the specificity of the primers. Moreover, as expected, the new primers were specific for *C. jejuni*, *C. coli*, *C. lari*, and *C. upsaliensis* strains. Figure 2 shows results obtained for some samples subjected to PCR.

**Figure 3:**
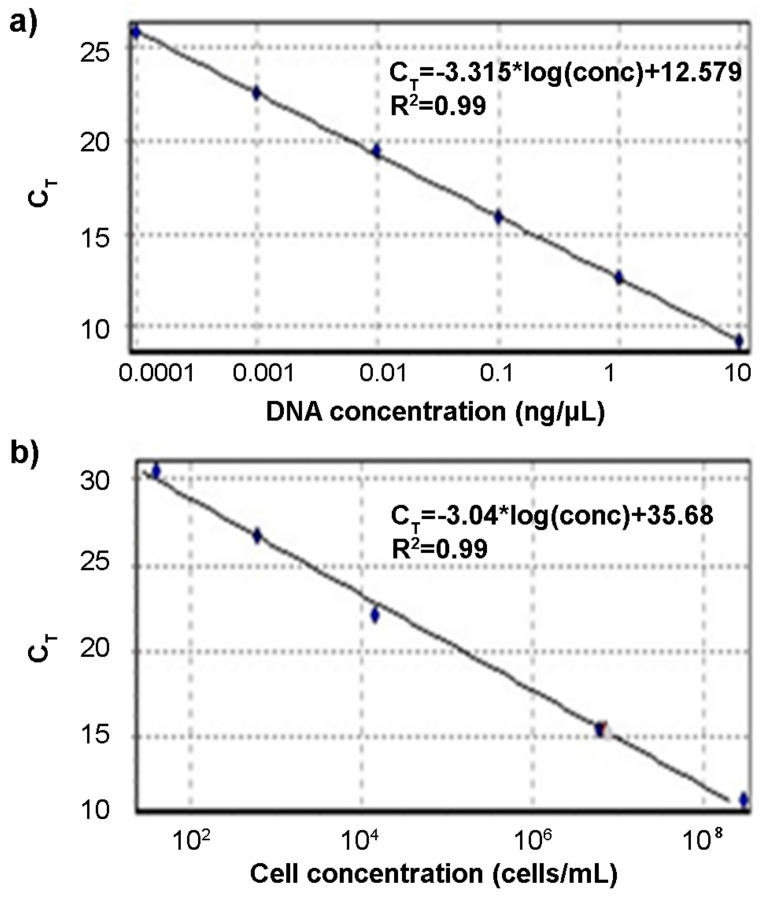
Calibration curves obtained using decimal dilution of DNA (**a**) and *Campylobacter* cells (**b**). (**a**) Standard curve of serial decimal dilutions of DNA of *Campylobacter jejuni* DSM 4688. The curve was obtained by plotting the threshold cycle (Ct) of each DNA dilution vs. the DNA concentration (ng/μL). (**b**) Standard curve of serial decimal dilutions of *C. jejuni* DSM 4688 cells. The curve was obtained by plotting the Ct of each cell dilution vs. the cell concentration of 2.09 × 10^8^, 7.38 × 10^6^, 2.66 × 10^4^, and 5.9 × 10^2^ cell/mL.

Primers were then tested in qPCR by using DNA extracted from *C. jejuni* DSM 4688. The calibration curve obtained using DNA dilutions from 10 ng/μL to 100 fg/μL (Figure 3a) showed a R^2^ of 0.99, slope of −3.315, and efficiency of 100.28%, indicating the high quality of primers. A calibration curve was also performed by using DNA extracted from serial decimal dilutions of *C. jejuni* DSM 4688 cells in the range from 10^8^ to 10 cell/mL (Figure 3b). The curve showed a R^2^ of 0.99, slope of −3.04, efficiency of 113%, and limit of detection of about 4.6 × 10^2^ cells/mL.

Table S3 reports the number of cells per milliliter evaluated by using DNA diluted at 1:1000. The cell concentrations extracted from the curve were confirmed by plate count evaluation performed on the same samples.

The qPCR assay was then applied on CC_t0_ and CC_t48_ samples. The obtained results are reported in Table 3. Five chicken samples (2 CC, 3 CC, 8 CC, 10 CC, and 17 CC) were positive for *Campylobacter* at t_0_ (before enrichment), and seven (2 CC, 3 CC, 8 CC, 9 CC, 10 CC, 16 CC, and 17 CC) at t_48_ (after enrichment).

CampyPFw and CampyPRv used in the qPCR assay enabled the detectection of *Campylobacter* DNA at concentrations as low as 100 fg/µL, while previously designed primers needed 10^3^ ng of DNA to provide results as reported by Alves [30]. Similarly, the limit of detection of 4.6 × 10^2^ cells/mL obtained using cell dilutions was lower than those previously reported by Wolffs et al. [31], Papic et al. [20], Alves et al. [30], and Wolffs et al. [32], which were 1 × 10^3^ CFU/mL, 2.6 × 10^3^ CFU/mL, 3 × 10^3^ CFU/mL, and 1.2 × 10^3^ CFU/mL, respectively. Improved sensitivity obtained by the new primers can be explained by the selected sequence used for primer annealing. CampyPFw and CampyPRv hybridized up to three points in the *Campylobacter* spp. sequence, as tested with BLAST producing three amplicons of 131–132 bp from 40,908 to 41,039, 396,123 to 396,254, and 700,253 to 700,384 on the DNA sequence CP040608.1 of *Campylobacter* spp. Moreover, CampyPFw and CampyPRv primers can be used for both PCR and qPCR in contrast to some previously published primers. For instance, Khan et al., [33] designed an efficient couple of primers that can be used only for PCR because their amplicon was too long for qPCR analysis.

The number of *Campylobacter* cells present in the positive samples was calculated by considering that 100 fg of DNA corresponds to approximately 50 cells [34] and by considering the value obtained by relating the length of the genome of a cell, which is about 1.6 × 10^6^ bp, to the weight of a base pair of 650 Daltons [35]. By hypothesizing that one cell of *Campylobacter* spp. contains 2 fg of genomic DNA, we refer the DNA value of samples 2 CS_t0_, 3 CS _t0_, 8 CS _t0_, 10 CS _t0_, and 17 CS _t0_ to about 2.45 × 10^3^, 5.84 × 10^3^, 4.64 × 10^3^, 1.20 × 10^3^, and 1.17 × 10^3^ cells/mL, respectively. *Campylobacter* was present in low numbers in meat samples tested when compared to bacteria such as coliforms and Enterobacteriaceae (Table 1). The high sensitivity and selectivity of primers enabled *Campylobacter* DNA detection in the presence of meat background bacteria. The data obtained indicate the usefulness of the primer probe set to detect and quantify *Campylobacter* in naturally contaminated chicken meat (Table 3).

Implementation of qPCR methods for the detection of *Campylobacter* in poultry meat requires optimization regarding both identification and quantification aspects. Although more than 85% of human campylobacteriosis is caused by *C. jejuni*, other *Campylobacter* strains isolated from poultry can also induce infections. The prevalence of *C. coli*, *C. lari*, and *C. upsaliensis* strains was found to be as high as 40%, 6%, and 2.5%, respectively, in *Campylobacter*-positive poultry samples [21,36,37,38,39]. The occurrence of non-*C. jejuni-C. coli* strains is probably even higher because alternative *Campylobacter* species count for about 10% of positive isolates [17]. qPCR protocols using new primer probe set designed in this work can simultaneously target the 16S-23S rDNA sequences of *C. jejuni*, *C. coli*, *C. lari*, and *C. upsaliensis* i.e., the most prevalent *Campylobacter* strains. Another important improvement is the enhanced sensitivity of detection obtained using the new primers, which enabled bacterial quantification in naturally contaminated chicken meat without an enrichment step. Most of the diagnostics available for the detection of *Campylobacter* are time-consuming and require the enrichment step, which is not adapted taking into account that chicken meat is consumed within only a few days after preparation. Adding enrichment to a PCR protocol improves the detection rate but impedes bacterial quantification. In addition, the enrichment medium may contain DNA polymerase inhibitors, which can markedly impair *Campylobacter* detection and quantification.

The results of the present study suggest that the new primer probe set may improve qPCR protocols for sensitive *Campylobacter* identification in poultry samples. Its implementation in daily routine analyses still requires validation on a large number of samples. It will be interesting to combine new primers with other optimized qPCR steps that include maximal removal of inhibitors from the matrix, utilization of inhibitor-resistant DNA polymerases, and automated DNA extraction procedure. We believe that in that way a robust qPCR protocol will be obtained for the reliable quantification of *Campylobacter* needed for surveillance programs to reduce contamination on chicken samples.

### 3.3. Sequencing

The sequences of amplicons obtained with CampyPFw and CampyPRv confirmed the specificity of the primers that detected *C. jejuni* in the analyzed samples with an identity of 99% and E-Value of zero. Sequencing also confirmed the prevalence of *C. jejuni* in chicken meat, which is in agreement with the published data [40,41,42].

## 4. Conclusions

CampyPFw and CampyPRv primers used in this study are specific and sensitive and can be used for real-time quantification of *Campylobacter* in naturally contaminated chicken samples. The qPCR protocol proposed is simple and rapid and could be directly used to examine chicken meat samples containing low bacterial titers. Since food contamination with *Campylobacter* is an important food safety concern, this assay can be a useful tool for detecting and monitoring the most prevalent *Campylobacter* species in contaminated foods.

This couple of primers able to detect the four most widespread *Campylobacter* species responsible for campylobacteriosis at levels below 10^3^ CFU/mL and in a amount of reduced time is a potential tool for improving food safety because samples contaminated al lower levels can be detected.

We believe that the sensitivity of the test can be further improved to lower numbers of CFU/g of *Campylobacter* spp. by using larger sample amounts (e.g., 25 g) in order to have a sufficient number of bacteria for detection despite their low absolute concentration, or it can be further improved by increasing the volume of DNA used as a template in PCR assays.

## Figures and Tables

**Table 1:** Microbial enumeration obtained for chicken samples of 10 g analyzed for total viable count, Enterobacteriaceae, coliforms, *E. coli*, yeasts, and molds expressed in Colony Forming Units (CFU)/g.

Samples	Total Viable Count	Enterobacteriaceae	Coliforms	*E. coli*	Yeasts	Molds
1 CC	9.9 × 10^7^	3.8 × 10^3^	2.0 × 10^3^	1.7 × 10^3^	2.3 × 10^4^	<50 *
2 CC	5.1 × 10^5^	1.1 × 10^3^	9.8 × 10^1^	2.0 × 10^2^	1.9 × 10^3^	<50 *
3 CC	1.9 × 10^5^	9.4 × 10^2^	1.3 × 10^1^	7.4 × 10^2^	7.8 × 10^2^	<50 *
4 CC	4.8 × 10^6^	1.2 × 10^4^	3.0 × 10^3^	5.3 × 10^3^	9.4 × 10^4^	<50 *
5 CC	9.5 × 10^6^	2.1 × 10^4^	7.0 × 10^3^	1.3 × 10^3^	6.3 × 10^5^	<50 *
6 CS	7.3 × 10^5^	1.7 × 10^3^	2.3 × 10^3^	7.6 × 10^2^	6.6 × 10^4^	<50 *
7 CC	9.6 × 10^5^	9.2 × 10^3^	6.4 × 10^3^	4.7 × 10^2^	3.3 × 10^4^	<50 *
8 CC	9.8 × 10^4^	5.2 × 10^3^	5.2 × 10^3^	3.2 × 10^2^	1.5 × 10^3^	<50 *
9 CC	1.8 × 10^5^	2.7 × 10^3^	1.4 × 10^3^	3.5 × 10^2^	2.2 × 10^3^	<50 *
10 CC	1.3 × 10^4^	1.5 × 10^3^	7.7 × 10^2^	8.3 × 10^1^	1.7 × 10^2^	<50 *
11 CC	2.1 × 10^6^	2.1 × 10^2^	4.2 × 10^1^	2.0 × 10^1^	6.1 × 10^3^	<50 *
12 CC	7.0 × 10^5^	2.6 × 10^3^	<50 *	8.0 × 10^2^	2.0 × 10^4^	<50 *
13 CC	1.2 × 10^7^	9.6 × 10^3^	9.5 × 10^1^	2.7 × 10^2^	3.5 × 10^4^	<50 *
14 CC	1.7 × 10^7^	2.4 × 10^3^	3.0 × 10^2^	0.6 × 10^3^	5.3 × 10^4^	<50 *
15 CC	3.7 × 10^7^	6.9 × 10^3^	6.5 × 10^1^	2.2 × 10^3^	4.5 × 10^4^	<50 *
16 CC	1.1 × 10^7^	1.6 × 10^4^	5.6 × 10^2^	2.6 × 10^2^	2.4 × 10^5^	<50 *
17 CC	<50 *	3.3 × 10^2^	<50 *	3.1 × 10^2^	<50 *	<50 *
18 CC	3.3 × 10^9^	2.1 × 10^4^	3.2 × 10^4^	6.0 × 10^2^	4.9 × 10^5^	<50 *
19 CC	3.8 × 10^9^	5.4 × 10^3^	1.3 × 10^4^	<50 *	3.9 × 10^5^	<50 *
20 CC	5.1 × 10^9^	2.4 × 10^4^	2.4 × 10^4^	1.5 × 10^2^	5.4 × 10^5^	<50 *

* Limit of detection of the method.

**Table 2:** Results of the ISO10272-1:2006 analyzing 10 g of chicken meat expressed as presence (+) or absence (−) of *Campylobacter* spp. by streaking on selective media mCCDA *, SKR °, and CAB ^§^ used after 4–6 h at 37 °C and 40–48 h at 41.5 °C and 25 °C. Oxidase and motility tests were performed on isolates.

Sample	mCCDA *	SKR ^°^	CAB ^§^	Confirmation Medium CAB		Oxidase	Motility
				41.5 °C, Aerobic	25 °C, Microaerophilic		
1 CC	+	−	+	+	−	+	−
2 CC	+	−	+	−	−	+	+
3 CC	+	−	+	−	−	+	+
4 CC	+	−	+	+	+	+	−
5 CC	+	−	+	+	+	+	−
6 CC	+	−	+	+	+	+	−
7 CC	+	−	+	+	+	+	−
8 CC	+	−	+	−	−	+	+
9 CC	+	+	+	+	+	+	−
10 CC	+	−	+	−	−	+	+
11 CC	−	−					
12 CC	−	−					
13 CC	−	−					
14 CC	−	−					
15 CC	−	−					
16 CC	+	+	+	−	−	+	+
17 CC	+	+	+	−	−	+	+
18 CC	+	+	+	+	+	+	−
19 CC	+	+	+	+	+	+	−
20 CC	+	+	+	+	+	+	−

* Modified Charcoal Cefoperazone Deoycholate Agar; ° Skirrow’s medium; ^§^ Columbia agar base.

**Table 3:** Chicken samples (CC) analysed by qPCR at t_0_ and at t_48_. Mean Ct values with standard deviation (SD); cell quantification expressed in cell/mL; and DNA quantification expressed in fg/µL.

CC Samples	t_0_			t_48_	
	Mean Ct ± DS	Cell/mL	DNA fg/uL	Mean Ct ± DS	DNA fg/uL
1 CC	27.55 ± 0.25	-	-	26.99 ± 0.16	-
2 CC	24.87 ± 0.10	3.60 × 10^3^	1.96 × 10^2^	13.19 ± 0.19	6.54 × 10^5^
3 CC	23.62 ± 0.38	9.27 × 10^3^	4.67 × 10^2^	12.97 ± 0.03	7.62 × 10^5^
4 CC	27.45 ± 0.22	-	-	27.40 ± 0.23	-
5 CC	28.1 ± 0.25	-	-	28.15 ± 0.17	
6 CC	28.3 ± 0.19	-	-	29.91 ± 0.09	
7 CC	28.58 ± 0.42	-	-	28.46 ± 0.23	
8 CC	23.95 ± 0.05	7.22 × 10^3^	3.71 × 10^2^	18.89 ± 0.10	1.25 × 10^4^
9 CC	27.3 ± 0.19	-	-	22.81 ± 0.18	8.20 × 10^2^
10 CC	25.91 ± 0.14	1.78 × 10^3^	9.59 × 10^1^	19.25 ± 0.25	9.72 × 10^3^
11 CC	27.94 ± 0.18			27.72 ± 0. 28	
12 CC	27.58 ± 0.23	-	-	26.48 ± 0.09	
13 CC	27.06 ± 0.10	-	-	26.68 ± 0.32	
14 CC	27.07 ± 0.18	-	-	26.82 ± 0.12	
15 CC	27.34 ± 0.25	-	-	26.74 ± 0.10	
16 CC	28.05 ± 0.15	-	-	25.98 ± 0.08	9.07 × 10^1^
17 CC	25.94 ± 0.32	1.60 × 10^3^	9.32 × 10^1^	22.62 ± 0.32	9.29 × 10^2^
18 CC	30.83 ± 0.39	-	-	27.33 ± 0.34	
19 CC	28.7 ± 0.31	-	-	26.96 ± 0.33	
20 CC	28.41 ± 0.35	-	-	27.10 ± 0.32	

## Data Availability

Not applicable.

## Supplementary Materials

The following are available online at https://www.mdpi.com/article/10.3390/foods10102341/s1, Table S1: Microorganisms used in the work; Table S2: Accession number of the sequences analyzed in silico to design primers CampyPFw and CampyPRv; Table S3: Data obtained with chicken meat samples artificially spiked with *Campylobacter jejuni*.
