# Supplementary material for: Enrichment Free qPCR for Rapid Identification and Quantification of Campylobacter jejuni, C. coli, C. lari, and C. upsaliensis in Chicken Meat Samples by a New Couple of Primers
Source: Foods. 2021 Sep 30;10(10):2341. doi: 10.3390/foods10102341 (PMC8535059; doi:10.3390/foods10102341)
Supplement: Supplementary file 1 [file foods-10-02341-s001.zip › foods-1390541-supplementary.pdf]

foods

MDPI

SUPPLEMENTARY INFORMATION

Enrichment Free qPCR for Rapid Identification and Quantification of Campylobacter jejuni, C. coli, C. lari and C. upsaliensis in Chicken Meat Samples by a New Couple of Primers

Priya Vizzini [1], Jasmina Vidic [2] and Marisa Manzano [1,*]

Dipartimento di Scienze AgroAlimentari, Ambientali e Animali, Università di Udine, 33100, Udine, Italia. vizzini.priya@spes.uniud.it (PV); marisa.manzano@uniud.it (MM).

2 Université Paris-Saclay, INRAE, AgroParisTech, Micalis Institute, 78350 Jouy en Josas, France. jasmina.vidic@inrae.fr (JV)

* Correspondence: marisa.manzano@uniud.it (Dipartimento di Scienze AgroAlimentari, Ambientali e Animali, via Sondrio 2/A, 33100 Udine, Italy)

Summary

1. Table S-1 reports the microorganisms used in the work.
2. Table S-2. reports the accession numbers of sequences used in the work.
3. Table S-3. rapports data obtained with chicken meat samples artificially spiked with *Campylobacter jejuni*

Table S-1. List of the microorganisms and DNAs used in the work.

	Microorganisms	Collection code
**Positive controls**	*Campylobacter jejuni subsp. jejuni*	DSM 4688[A]
	C. coli	DSM 24155[A]
	C. lari subsp.lari	DSM 11375[A]
	C. upsaliensis	DSM 5365[A]
**Negative controls**	*C. fetus*	DSM 5361[A]
	C. cryaerophila	DSM 7289[A]
	Helicobacter pylori DNA	DSM 7492[B]
	H. pylori	ICS [C]
	H. suis	DSM 19735[A]
	Arcobacter butzleri DNA	DSM 8739[B]
	Listeria monocytogenes	ATCC 7644[D]
	L. innocua	DSM 20649[A]

L. seeligeri	DSM 20751[A]
L. marthii	DSM 23813[A]
L. welshimeri	DSM 15452[A]
L. ivanovii	DSM 52491[A]
Staphylococcus aureus	DI4A[E]
Bacillus cereus	DSM 4282[A]
B. cereus	DI4A RC3[E]
B. subtilis	DSM 4181[A]
Salmonella enterica	DSM 9378[A]
Escherichia coli	DISTAM[F]
Lactobacillus plantarum	ATCC RAA 793[D]
Saccharomyces cerevisiae	ATCC 36024[D]

[A]DSM: Deutsche Sammlung von Mikroorganism und Zellkulturen GmbH (Braunschweigh, Germany); [B] DNAs from DSM collection; [c]ICS: Isolated from Clinical samples (Hospital of Udine, Italy); [D]ATCC: American Type Culture Collection (Manassas, VA, USA); [E]DI4A: Department of Agricultural, Food, Environmental and Animal Sciences (Udine, Italy); [F] DISTAM: Department of Food technologies and Microbiology Science (Milan, Italy).

Table S-2. Accession number of the sequences analyzed in silico to design CampyPFW and CampyPRV primers.

	BACTERIA SEQUENCES (16S and 16S-23S ribosomal RNA gene)				
N	Accession number	Genus, species	N.	Accession number	Genus, species
1	EF373994.1	*Aeromonas sobria*	15	AB089244.1	*Morganella morganii*
2	AM062666.1	*Bacillus cereus*	16	AF405374.1	*Pediococcus pentosaceus*
3	EF205020.1	*B. subtilis*	17	FJ518598.1	*Proteus vulgaris*
4	AF047423.1	*Citrobacter freundii*	18	JN418884.1	*Pseudomonas aeruginosa*
5	EF527445.1	*Escherichia coli*	19	AF268968.1	*Ps. brennerii*
6	FJ410387.1	*Enterobacter aerogenes*	20	AM086254.1	*Ps. brennerii*
7	AF047426.1	*E. aerogenes region*	21	EF198908.1	*Ps. fluorescens*
8	EU078570.1	*E.cloacae*	22	KF857261.1	*P. migulae*
9	AY277975.1	*Helicobacter ganmani*	23	AF046822.1	*Salmonella enterica*
10	DQ399570.1	*Klebsiella pneumoniae*	24	KX696458.1	*Shigella sonnei*
11	AB092638.1	*Lactobacillus plantarum*	25	U11784.1	*Staphylococcus aureus*
12	AF000655.1	*Legionella pneumophila*	26	AY531067.1	*Vibrio parahaemolyticus*
13	AB295116.1	*Leuconostoc lactis*	27	FJ429988.1	*Weissella cibaria*
14	AY684791.1	*Listeria monocytogenes*	28	AF293850.1	*Yersinia enterocolitica*

**BACTERIAL WHOLE DNA**					
N.	Accession number	Genus, species	N.	Accession number	Genus, species
1	kv861263.1	*Aeromonas sobria*	16	lvwz01000001.1	*Pseudomonas brenneri*

2	ap007209.1	*Bacillus cereus*	17	NZ_LDET01000015.1	*Ps. fluorescens*
3	nz_cp011534.1	*Bacillus subtilis*	18	fnty01000001.1	*Ps. migulae*
4	CP016762.1	*Citrobacter freundii*	19	acfl01000033.1	*Saccharomyces cerevisiae*
5	fm991728.1	*Helicobacter pylori b38*	20	NC_003197.2	*Salmonella enterica subsp. Enterica*
6	ba000007.2	*Escherichia coli O157:H7*	21	nz_lfwb01000312.1	*Morganella morganii*
7	NZ_LYDO01000004.1	*Enterobacter aerogenes*	22	nz_lyem01000481.1	*Shigella sonnei*
8	nz_cp011798.1	*E. cloacae*	23	ap008934.1	*Staphylococcus saprophyticus subsp. saprophyticus*
9	nz_cp010435.1	*Helicobacter pylori*	24	kn150745.1	*Proteus vulgaris strain*
10	fo203501.1	*Klebsiella pneumoniae*	25	nz_lirr01000034.1	*Vibrio parahaemolyticus*
11	nz_cp012650.1	*Lactobacillus plantarum*	26	nz_cp012873.1	*Weissella cibaria*
12	nz_cp011105.1	*Legionella pneumophila*	27	am286415.1	*Yersinia enterocolitica subsp. enterocolitica*
13	nz_cp011105.1	*Legionella pneumophila*	28	cp007224.1	*Pseudomonas aeruginosa*
14	ae005176.1	*Lactococcus lactis subsp. lactis*	29	cp015918.1	*Pediococcus pentosaceus*
15	nz_bbrp01000009.1	*Listeria monocytogenes*			

***CAMPYLOBATCER* SPP. (16S and 16S-23S gene)**

N.	Accession number	Genus, species
1	KP064555.1	*Campylobacter fetus*
2	GQ167709.1	*C. concisus*
3	JX912515.1	*C. gracilis*
4	L04322.1	*C. concisus,*
5	AB301966.1	*C. fetus subsp. fetus*
6	M65011.1	*C. fetus subsp. venerealis*
7	NZ_FPBB01000005.1	*C. hyointestinalis*
8	nz_jhqq01000009.1	*C. mucosalis*
9	nz_jmti01000045.1	*C. sputorum biovar sputorum*

ANIMALS (16S gene)

N.	Accession number	Genus, species
1	KP721213.1	*Sus scrofa domesticus*
2	DQ334849.1	*Meleagris gallopavo*
3	AB489247.1	*Gallus gallus*

4. Section SI3 reports an additional test acrried out on four chicekn meat samples.

Anlysis of hicken meat samples artificially spiked with *Campylobacter jejuni*

Serial decimal dilutions of *C. jejuni* overnight culture (BHI, microaerophilic conditions) containing approximately 10^8 cells/mL, were inoculated into meat samples (named SC) to reach final concentrations of 10^7, 10^5, 10^3, and 0 cell/g of meat. A plate count was performed on the spiked samples to obtain the correspondent CFU/g value. DNA of SCs was extracted at t_0 from the homogenization Stomacher bags as reported in Section 2.3 of Materials a Methods and used for qPCR. Results of the analyses are reported in the Table S-3 below.

Table S-3. Mean Ct values with standard deviation (SD), cell quantification expressed in cell/mL and by plate count method evaluation (CFU/g).

SC Samples		qPCR	Plate count method (mCCDA)
	**mean Ct ± SD**	**cells/ mL***	**CFU/g**
1 SC	21.37 ± 0.47	5.10E+04	1.16×10^{7}
2 SC	26.50 ± 0.19	1.05E+03	1.07×10^{5}
3 SC	32.16± 1.16	1.43E+01	1.22×10^{3}
4 SC	32.40 ± 0.29	1.20E+01	< 20

* DNA used as a template in the test was diluted 1:1000
